# Focus groups on digital cognitive assessment in the context of Alzheimer's disease

**DOI:** 10.1177/20552076251318903

**Published:** 2025-02-12

**Authors:** Sophie M van der Landen, Rosanne L van den Berg, Matthijs J Keijzer, Mariska N van Liere, Casper de Boer, Leonie N C Visser, Wiesje M van der Flier, Hanneke F M Rhodius-Meester, Sietske A M Sikkes

**Affiliations:** 1 685848 Alzheimer Center Amsterdam, Neurology, Amsterdam University Medical Center, Amsterdam, The Netherlands; 2 601873 Amsterdam Neuroscience, Amsterdam, The Netherlands; 3 416668 Department of Clinical, Neuro and Developmental Psychology, Faculty of Movement and Behavioral Sciences, 1190 Vrije Universiteit Amsterdam, Amsterdam, The Netherlands; 4 596712 Neurocast BV, Amsterdam, The Netherlands; 5 Division of Clinical Geriatrics, Center for Alzheimer Research, Department of Neurobiology, Care Sciences and Society, 27106 Karolinska Institutet, Solna, Sweden; 6 Medical Psychology, Amsterdam University Medical Center, Amsterdam, The Netherlands; 7 Amsterdam Public Health, Quality of Care, Personalized Medicine, Amsterdam, The Netherlands; 8 567874 Department of Epidemiology and Data Science, 1190 Vrije Universiteit Amsterdam, Amsterdam University Medical Center, Amsterdam, The Netherlands; 9 Geriatric Medicine, The Memory Clinic, 155272 Oslo University Hospital, Oslo, Norway

**Keywords:** Digital cognitive assessment, Alzheimer's disease, early disease detection, end user perspectives, patient perspectives, focus groups

## Abstract

**Introduction:**

Digital cognitive assessments (DCAs) may facilitate early recognition of cognitive decline in the context of Alzheimer's disease (AD). While DCAs are increasingly emerging, they are often not used in practice. We assessed facilitators and barriers of using DCAs according to older individuals and patients.

**Methods:**

In five focus groups, we presented three different DCAs to older individuals with unimpaired cognition (*n* = 14), subjective cognitive decline (*n* = 11) and mild cognitive impairment with biomarker-confirmed AD (*n* = 4) and their caregivers (*n* = 2). Participants discussed facilitators and barriers that could influence the use of DCAs. Focus groups were recorded, and transcripts were analysed using thematic analysis.

**Results:**

Three main themes were identified: (1) test motivation (‘Do I want to know my brain health?’), facilitated by early disease recognition, while impeded by fear of dementia; (2) digital test suitability (‘Do I want to use a *digital* test?’), enabling at-home testing, while lacking personal contact; and (3) digital test characteristics (‘What makes a digital test a good test for me?’), where user-friendliness was emphasized as a key facilitator to overcome digital incompetence.

**Discussion:**

Participants recognized the added value of DCAs, and multiple factors were identified as facilitators and barriers for their use. Similar factors could be a barrier for one, yet facilitated others, underlining the need for a personalized approach. Strategies to minimize barriers and exploit facilitators would ultimately foster implementation.

## Introduction

Cognitive testing is a central aspect of the diagnostic work-up for Alzheimer's disease (AD).^1^ AD is the most common cause of dementia, and the number of patients with AD is rapidly increasing with an expected prevalence of 152 million patients in 2050.^2^ At the same time, the emergence of biological biomarkers and technological advances facilitates earlier disease recognition, which opens a window for future interventions and new disease-modifying therapies that are on the horizon.^3–5^ To date, however, most AD patients are not diagnosed until the mild-to-moderate dementia stage.^2^ In addition, up to approximately 60% of dementia cases even remains undiagnosed.^6,7^ This has multiple explanations, such as limited access to diagnostic services, long waiting lists and time-consuming tests.^3^ New, scalable, timesaving and easy to implement methods for early recognition of cognitive decline in AD are thus needed.

Tackling these challenges, digital cognitive assessments (DCAs) could play a key part in the clinical care pathway of the future.^2,8^ These assessments could be implemented within different contexts of use, from reassuring the worried-well, to screening, diagnosing and monitoring of patients. A variety of digital assessments, sometimes referred to as cognitive biomarkers, is being developed, ranging from digitalized versions of existing paper-and-pencil tests, to novel approaches including eye-tracking, virtual reality and spoken language analysis.^9^ Although DCAs are still in the early phases of development, there is some promising evidence for initial clinical and biological validation of DCAs in early-stage AD.^9–11^ Digital assessments have several advantages when compared to more traditional paper-and-pencil tests. They, for example, enable large-scale self-administration at home, ensure standardized testing and scoring, generate refined and objective outcomes and pave the way to more sensitive measurement of (cognitive) change.^9^

While healthcare professionals recognize the value of DCAs, they are hardly implemented.^12–15^ Towards implementation, one of the key elements is identification of implementation barriers and facilitators from different stakeholders including older individuals and patients as end users.^14,16^ The involvement of patient perspectives ensures that clinical innovations such as DCAs will meet their needs and preferences, which may facilitate the translation from development to implementation. Previous work showed that patients and care partners have a positive attitude towards digital tools in general, being motivated by, for example, user-friendliness, trustworthiness, timesaving and personalization, although barriers were also raised, including lack of personal contact and difficulties with self-administration.^17–19^ However, it is currently unknown whether the same factors also apply to DCAs according to older individuals in different places in the patient journey, i.e. from cognitively healthy, to being worried and visiting the memory clinic, and being diagnosed. In this realm, older individuals in early AD stages represent the most relevant potential end user group since cognitive assessments are of the greatest need and hold the highest potential for benefit.

In this study, we assessed the facilitators and barriers for the use of DCAs by considering three different use cases of DCAs (i.e. digitalized paper-and-pencil assessment, speech analysis, smartphone typing behaviour) that are currently under development or validation. Specifically, we evaluated the perspectives of cognitively unimpaired (CU) individuals of older age, individuals with subjective cognitive decline (SCD) and individuals with mild cognitive impairment (MCI) and their care partners. Identifying these factors could ultimately help to bridge the translational gap between development and implementation of DCAs for different disease stages.

## Methods

### Study design and ethics statement

In this qualitative study, we followed an inductive (bottom-up) approach to identify facilitators and barriers for using DCAs and conducted focus group sessions between June and September 2023. All focus groups were conducted in the outpatient clinic of Alzheimer Center Amsterdam in the Amsterdam University Medical Center (UMC). The study was reviewed by the board of the Medical Ethics Committee of the VU University Medical Center, Amsterdam UMC. All participants provided written informed consent before participation according to the Declaration of Helsinki.

### Participants

We set out to conduct focus groups consisting of a maximum of eight participants per group according to methodological guidelines for focus groups^20^: at least one focus group with CU individuals, one with individuals with SCD and one with individuals with MCI and their caregivers. We recruited participants either by flyers where participants were invited to sign up via email, or they were directly approached by researchers via email or by phone. Participants were recruited from four sources: (1) the memory clinic-based Amsterdam Dementia Cohort (ADC),^21^ (2) the memory clinic-based SCIENCe cohort of individuals with SCD,^22^ (3) population-based registry hersenonderzoek.nl^23^ and (4) a population-based Dutch elderly organization ‘Katholieke Bond van Ouderen Protestants Christelijke Ouderenbond (KBO-PCOB)’. For those included via ADC and SCIENCe, a syndrome diagnosis of SCD or MCI due to AD was made in clinical consensus using published diagnostic criteria.^24,25^ For individuals with MCI, AD pathology was confirmed by use of biomarkers (visual reading of amyloid positron emission tomography scan^26^ or the ratio ptau181/Aβ42 > 0.020 in cerebrospinal fluid.^27^ CU participants were recruited via hersenonderzoek.nl and the KBO-PCOB. All participants had to be able to read and speak Dutch.

In total, we conducted five focus groups with 31 participants. Since >8 eligible CU individuals and individuals with SCD wanted to participate, we held two focus groups with CU individuals (*n*_group 1 _= 6, *n*_group 2 _= 8), two focus groups with individuals with SCD (*n*_group 3 _= 6, *n*_group 4 _= 5) and one focus group with individuals with MCI and their care partners (*n*_group 5 _= 6). After these five focus groups, data saturation was reached (see ‘Analysis’ section). Table 1 shows an overview of participant characteristics per diagnostic group. On average, participants were 70 ± 6 years (range: 58–85), 19 (61%) were female and the mean years of education was 12 ± 3 (range: 8–17).

**Table 1.** Participant characteristics.

	**Total**	**CU**	**SCD**	**MCI**	**Care Partners**
***N***	31	14	11	4	2
**Age (years), M ± SD**	70 ± 6	69 ± 7	71 ± 5	69 ± 3	N/A
**Female, *n* (%)**	19 (61%)	11 (79%)	7 (64%)	1 (25%)	2 (100%)
**Education (years), M ± SD**	12 ± 3	13 ± 3	13 ± 2	10 ± 0	N/A

*Note*. CU = cognitively unimpaired; SCD = subjective cognitive decline; MCI = mild cognitive impairment; SD = standard deviation; N/A = not available, as age and education level of the care partners were not asked.

### Procedures

Each focus group was led by two moderators, rotating between MK, RB, SL, and an assistant (ML), and designed to last approximately two hours. The moderators had no prior relationship with the participants. Two of the moderators were trained in conducting qualitative research, and subsequently trained the third moderator. Focus groups were recorded using the Olympus digital voice recorder VN-731PC and the recordings were saved in a secured environment of the Amsterdam UMC.

**Figure 1.**
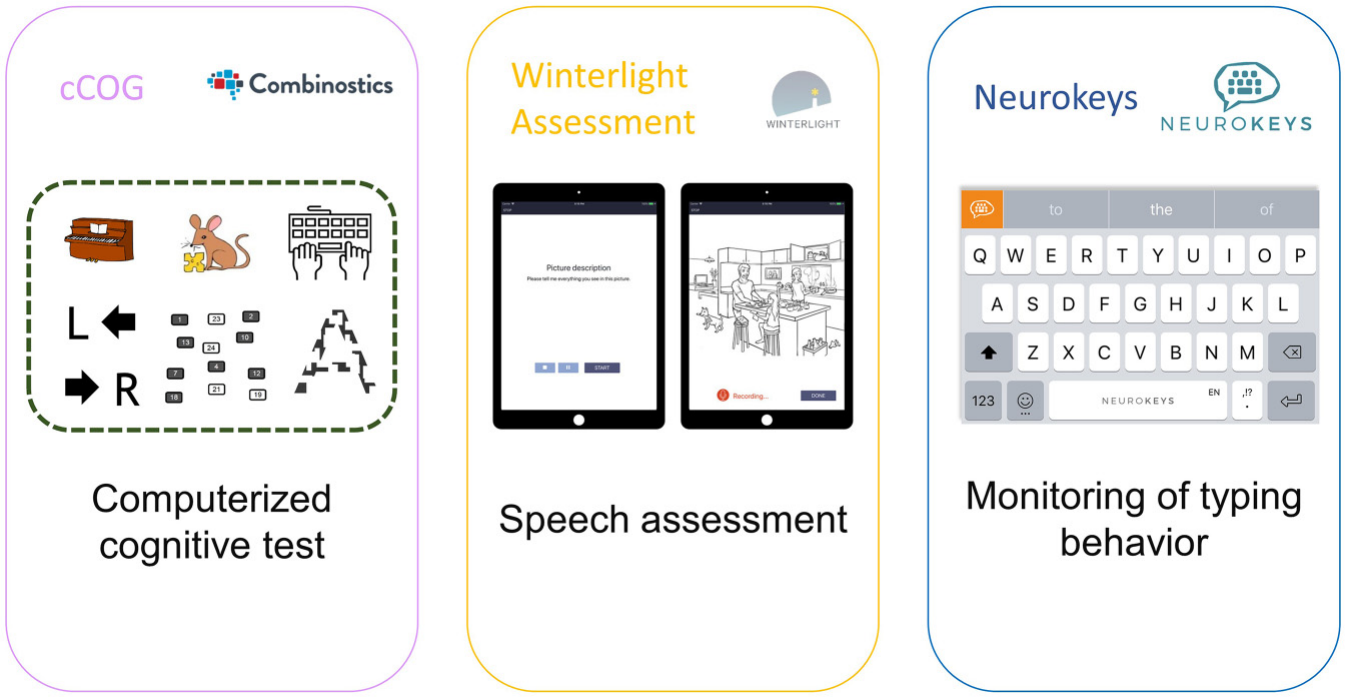
Overview of digital cognitive assessments presented as examples during the focus groups.

During an introduction round, the moderators were introduced as researchers in the field of DCAs. To ensure the use of consistent terminology in discussions during the focus group, a short presentation was given by the researcher leading the group. First, the definitions of AD and dementia were explained, and differences between paper-and-pencil tests and DCAs were described. To concretize the concept of DCA we presented participants with three examples of different types of DCAs (Figure 1). These comprised (A) cCOG,^28^ an active test including tasks such as recognizing fragmented letters and word list learning; (B) the Winterlight Assessment,^29^ a speech assessment that records speech for example during picture descriptions; and (C) Neurokeys,^30^ a passive measurement assessing everyday typing behaviour on a smartphone (e.g. key strokes). After this introduction, participants were invited to take part in the discussion about facilitators and barriers for using a DCA. To facilitate the discussion, green and red post-its were provided, and participants were instructed to individually write down factors that would hinder or stimulate them to use DCAs such as the ones presented. Thereafter, the differences and similarities of the identified barriers and facilitators were discussed in pairs. Afterwards, all facilitators and barriers were collected and elaborated upon in a group discussion, to gain more insight in the reasoning behind these factors. The focus group procedure and questions were pilot-tested in a group of colleagues with specific expertise in cognitive testing, including neuropsychologists, neuroscientists, clinicians and researchers. Based on this initial pilot-test, the focus group procedure was refined. Further adaptations to the procedure took place after the first focus group. Initially, we aimed to collect the post-its with facilitators and barriers on a single sheet, but as we noticed this was not practical and distorted the flow of the discussion, we further refined the focus group procedure by omitting this step in the following focus groups. The focus group procedure is visualized in Supplementary Figure 1.

### Analysis

The data were analysed using MAXQDA software,^31^ following the phases of thematic analysis by Clarke and Braun.^32^ First, the focus group audio recordings were transcribed verbatim, by ML and SL. Second, two authors, SL and RB, independently generated initial codes for the first transcript, which were derived bottom-up from the data. A meeting was held to discuss these codes and come to agreement. Coding of the remaining transcripts was then divided by SL and RB. After coding five focus groups, these authors noted that there were mainly recurring codes and agreed that data saturation was reached, and therefore no additional focus groups were necessary. Thereafter, overarching themes and sub-themes were derived from the initial codes during a consensus meeting. Finally, in an iterative process of translating, renaming and reviewing themes by four of the authors (SL, RB, WF, and SS), a final agreement was reached on the themes and subthemes. Thereafter, the identified subthemes were narratively compared across diagnostic groups.

## Results

**Figure 2.**
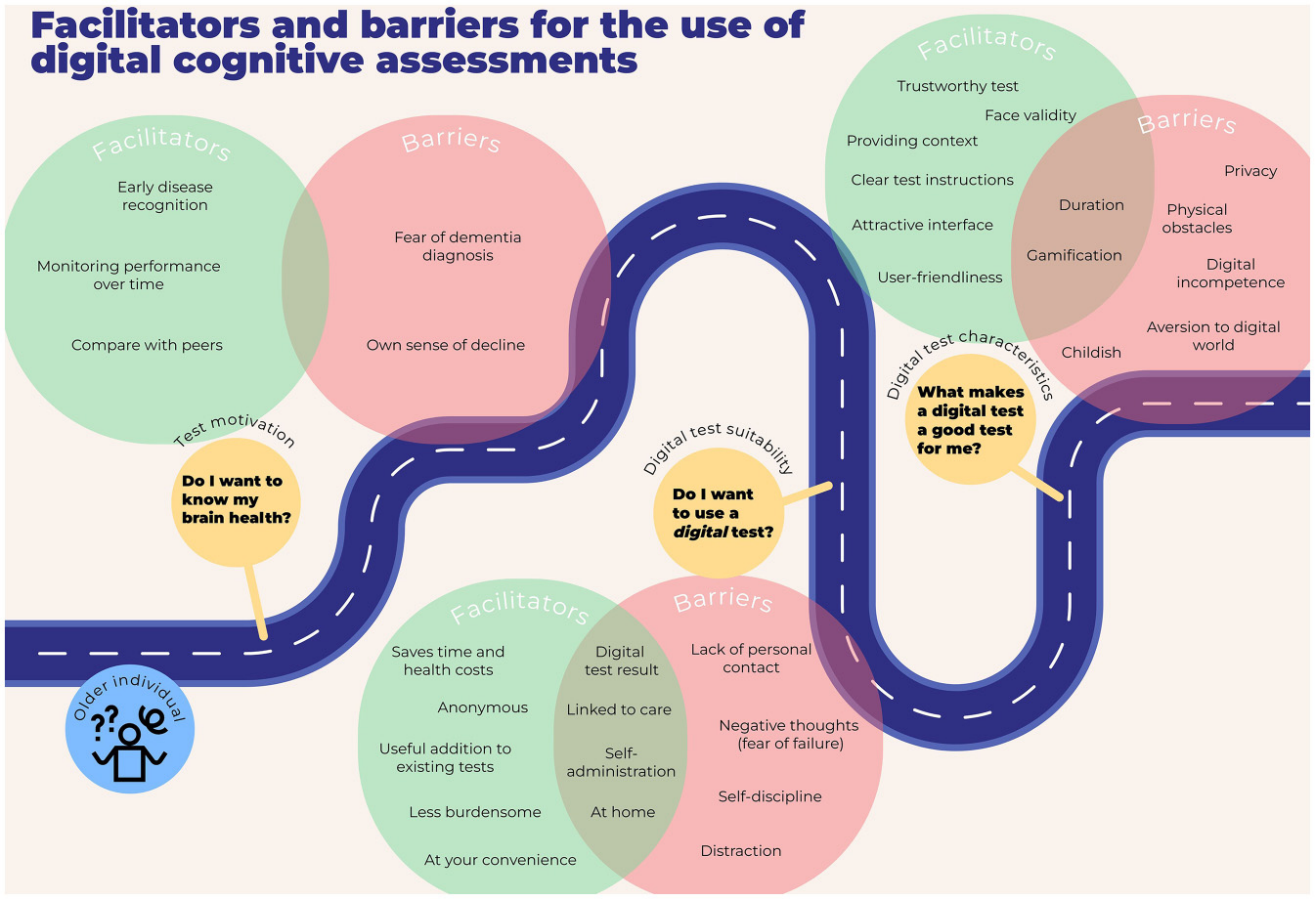
Facilitators and barriers for the use of digital cognitive assessments.

The data showed three overarching main themes that followed the sequence of patients’ questions along the diagnostic journey, as depicted in Figure 2. The identified themes were (1) test motivation (‘Do I want to know my brain health?’); (2) digital test suitability (‘Do I want to use a *digital* test?’); and 3) digital test characteristics (‘What makes a digital test a good test for me?’). Within each main theme several subthemes were identified, which revealed large variation in personal preferences, as evidenced by identification of variable and contradictory barriers and facilitators between and within diagnostic groups. The three main themes and their sub-themes are described in the following three subsections. Subthemes are marked in bold in the text below. In Supplementary Table 1, an overview is provided of the identified themes per diagnostic group.

### Test motivation

Focus group results concerning the question ‘Do I want to know my brain health?’ indicated that test motivation was on the one hand facilitated by the prospect of **early disease recognition**, which was mentioned in the CU and SCD groups:

Well, it’d be a motivator for me for sure, wanting to get an early diagnosis. (CU)

On the other hand, concerns about early disease recognition were also raised:

The fear of the big moment. So that's my big question: what's the point of finding out at such an early stage? (CU)

Related to the concerns of early disease recognition, for all diagnostic groups the willingness to test cognition was impeded by the **fear of a dementia diagnosis**. Participants indicated that using DCAs would raise awareness on their brain health, thereby increasing their worries, which would impede test motivation.

I’m afraid of the result, and underlying that is my fear of dementia. (CU)

Moreover, the ability to **monitor cognitive performance** over time motivated the willingness to test. It was mentioned that participants would like to know if their cognition declined:

The first point I came up with: the one thing that mustn’t happen is for me to end up in some room in diapers; that's not going to happen. I’ve already had my wishes about all that documented, so now I constantly want to know how far I am. (MCI and caregivers)

In contrast, in the SCD group test willingness was motivated by monitoring cognitive improvement. For others the desire for a confirmation that cognition is still within normal ranges was expressed. In the SCD group, it was specifically mentioned that **comparing performance** or decline with that of **peers **was preferable:

How do people of my age perform who haven’t got Alzheimer's? That's what you want to know. Is this normal, is it just old age – something everyone has – or is it oh no, this is quite different to other people of that age? (SCD)

In contrast, some participants indicated no need for a (digital) test to measure their cognitive abilities, as they would have their **own sense of cognitive decline** themselves:

I’ll be able to tell if I’m getting worse; I don’t really need an app for that. (SCD)

Yes, I’m very clear that I shouldn’t need to know. I think you’d notice if you … if you were getting worse. But I’d rather not know, no. (MCI and caregivers)

### Digital test suitability

During the focus groups participants discussed topics linked to the question ‘Do I want to use a *digital* test?’, where they elaborated upon whether they perceived digital tests as an appropriate measure to assess their cognition, when compared to paper-and-pencil tests. CU and SCD participants mentioned that digital tests would be a **useful addition to current clinical practice**:

I thought it was a good idea from the start and I also think it's geared to the future, we can’t not do this, it's simply the future. And yes, sure, the doctors will definitely get more time for their own specialism because those results are a determining factor. But even so, there should still be a normal examination. (SCD)

Digital tests enable **self-administration** of cognitive performance, which was perceived as both a facilitator (CU and SCD) and a barrier (all groups) for the willingness to use DCAs. CU individuals who perceived it as a motivator mentioned that they would prefer testing **anonymously**, without a** link to medical care **as an aid to decide whether or not to seek medical help:

Well, if I go and get this done through the family doctor, it would count as a medical examination, but if I do it at home kind of as a check on my cognitive health out of interest, then I’d definitely want to do that anonymously. (CU)

In contrast, for other CU and SCD participants a **lack of personal contact** was perceived as a barrier to self-administered digital testing. In the absence of personal contact, there might be no possibility to ask clarifying questions, or to express feelings of distress. Especially for individuals who would experience a **fear of failure**, performing the test in the presence of a health care professional may be preferable for guidance and comfort.

It reminds me a bit of […] a school test – soon I’ll look dumb, oh no, I’m getting that marked test feeling, mainly a fear of failure. So who can I discuss this with? (CU)

Particularly in the event that test results would be indicative of cognitive impairment, participants indicated to prefer being guided by a health care professional:

So say the test says: that's it, you’ve got signs of dementia, I think I’d feel pretty lonesome there with my iPad. That's not something I like the sound of. (CU)

A theme linked to (a lack of) personal contact when using DCAs considered receiving a **digital test result**. Participants in all groups differed in their preferences regarding receiving the digital test result directly via the DCA themselves, or through communication by a health care professional. It was suggested that digital test results could be supplemented by the advice to contact or not to contact a health care professional, such as a general practitioner. Depending on the results, users could then decide for themselves to seek medical help as a next step:

And if I get the result and have doubts, then I’d see my family doctor. (CU)

Based on the diverging perspectives on direct disclosure of the results, participants recommended to tailor it to personal preferences whether the results will be disclosed by the DCA or not:

You have that choice in your care file too: do you want to be able to see a certain result or not if you haven’t yet discussed it with your doctor? I can imagine that would be nice if you could build that into the app – do you want the feedback or not? (SCD)

Another aspect of digital testing is that it enables **at-home testing**, for which both practical advantages and disadvantages were raised. At-home testing requires a sense of **self-discipline**, for which two barriers were mentioned in all groups. First, if digital tests can be self-administered in the participant's home environment on a self-decided time and day, participants indicated to be worried about their discipline to remember to complete the assessment, or to finish the entire assessment:

You need to have the self-discipline, to remember things and to want to remember them, which I’m not so great at. I think I’d find that difficult. (MCI and caregivers)

I’d rather go to the hospital because then I’d go, I’d take the time for it instead of spending maybe a quarter hour and then thinking that's enough, bye! Whereas then you’d be coming specifically for it. I’d really need to concentrate and take my time with it, otherwise – knowing me – it would be OK for two weeks but by the third week I’d lose interest. (SCD)

A second factor related to self-discipline regarded integrity and cheating. Some participants responded that they would honestly complete self-administered tests, as they would be intrinsically motivated to test their cognition. Other participants, however, indicated the opportunity to write down words during a digital word learning task, or to ask a caregiver for help, or even to complete the test. Thus, it should be ensured that the patient is the one who is completing the task truthfully. Unfaithful test completion may for instance be supported by the fear of dementia:

You should build in a check so you know for certain it's the same person every time filling it in. Because I know from my own experience that, let's say, someone who definitely doesn’t have problems will just fill it in for that person, because they’d rather not know Dad is getting worse. (CU)

Other barriers regarding at-home testing were raised in all groups, involving **distractions** from the testing device or the home environment, both being a potential threat for reliability:

Because of course they [ads] are also irritating and incredibly distracting in apps like this. (MCI and caregivers)

But you don’t have the peace and quiet of a hospital where you’re alone and concentrating on that, with nothing going on around you. No doorbell, no phone, no people moaning, cats, you name it. (SCD)

In contrast, for other participants, the potential of remote testing in the participant's home environment would motivate them to use DCAs. For instance, in all groups an advantage of at-home testing was mentioned that tests can be performed **at your own convenience**. Another motivator related to at-home testing, that was raised by CU and SCD participants, was that at-home testing can be perceived as **less burdensome** and less intrusive:

And not having to come along, because coming there's something I find, um, not so pleasant and I also find coming here [to the hospital] difficult to cope with, to be honest. (SCD)

Moreover, in the SCD group, **saving time and health costs** was mentioned as an additional advantage of completing DCAs at home. For instance, such tests could replace (part of) the examination that is usually performed by a health care professional in a clinical setting.

…and especially reducing the doctor's working hours. Because if this was possible, […] and one test takes five minutes and the next twenty minutes, well then I don’t need someone in front of me for the whole day, someone who's badly underpaid at that. (SCD)

### Digital test characteristics

Third, we found topics related to the overarching question ‘What makes a digital test a good test for me?’. In all groups, **user-friendliness** was raised as an important test characteristic. Accordingly, a digital test should be easily accessible (e.g. available in an app store), easy to use and have an **attractive interface**. To facilitate this, it was discussed in the CU and MCI groups to make use of games and challenges, where for example different levels could be implemented. Not everyone was positive about **gamification **however, as this could be perceived as **childish**, while your brain health concerns a serious topic:

Don’t make it too childish, […] but we’re not little children. Right, that's how I see it, it's not a game, how to deal with your own death. That's what I reckon. (MCI and caregivers)

Again, contradictive preferences underline the need for the possibility to personalize, both in the way the DCA is presented as in the degree of difficulty of the tasks. Personal preferences were also identified concerning overall **test duration**. In all groups, preferences for test duration and the testing period (e.g. one day, a week or months) differed among individuals. Some participants indicated to prefer one long test rather than being monitored for a longer timeframe:

But that also makes me nervous. I know already that if I had to do that test every month, I’d be thinking about it so much during those days that it would affect my behavior, I guess. That's why I think once a year is more relaxing. (SCD)

Others indicated a preference for continuous testing or multiple short assessments over days, as long tests would not be feasible to complete in one go:

Yes, well, I thought five minutes would be fine, […] twenty minutes in one go might be a bit too long, so perhaps it would be better to split it into two ten-minute sessions. (MCI and caregivers)

I find that [how long it takes] an important detail, right, how long you have to spend concentrating on it, mainly to make sure you have enough peace and quiet. (SCD)

Irrespective of the duration, participants agreed that it should be clearly communicated what the test duration is, and for how long data will be collected, which could be a single moment, or multiple times in case of repeated testing paradigms or (passive) monitoring over time:

That would also help, if you know it's a week or it's two days. (CU)

In general, the importance of **clear test instructions** was emphasized as a facilitator by CU and SCD participants:

But the key thing is to have good instructions for these programs. The baseline you start from is so important anyway. (SCD)

Clear test instructions are especially relevant with regards to **digital incompetence**, which was mentioned in all groups as a challenge in the use of DCAs:

But I didn’t mean the apps for young people, I meant the younger generation who will be getting old soon; they’re used to cellphones but of course we didn’t grow up with cellphones, and particularly not with apps. (SCD)

Some participants across all groups even expressed a general **aversion to the digital world**:

I’ve never been interested in that at all, in digital stuff. I had to get to grips with it but it's not something that interests me at all. (MCI and caregivers)

This aversion to the digital world can come with feelings of concern regarding **privacy**, as raised by CU and SCD participants. It can be distressing to have the feeling of being out of control regarding where your personal data ends, and what your personal data is used for, which was identified as a barrier:

The feeling that in the background, right, the feeling that you’re being watched in the background and you don’t have any control over it any more. (CU)

In this context, information about **trustworthiness **would facilitate the use of DCAs in the CU and SCD groups. For example, it is of importance from which institution the test originates, e.g. an invitation from a renowned hospital is perceived as trustworthy:

It does make a difference whether it comes, for example, from the Alzheimer's Institute […]. But if there's a company behind it, a private party or something like that, then I won’t continue with it. (SCD)

Moreover, participants in the CU and SCD groups indicated worries regarding the **face validity** of digital tests:

If you’re making more typing errors, would that really be due to dementia? Or if you spell words wrong when you’re going fast. (SCD)

Right, I just don’t see the point in it. (CU)

A possible threat to face validity was suggested in CU and SCD groups to be **physical obstacles**, which should thus be taken into account with an adaptive version for individuals with motor or visual problems:

I also find the phone difficult sometimes. We were just talking about that: I prefer to look at the computer, as it were, because that's got a bigger screen. A phone is always real small and that can be handy for some things, but well, it can also be tricky sometimes. (SCD)

Participants in the CU and SCD groups proposed the possibility to** provide context** if there are factors that might have an influence on the results, such as physical obstacles or a bad night of sleep:

Sometimes it's like your head is stuffed with sawdust, you’ve slept badly or whatever… (CU)

Another useful option regarding the possibility of providing context would be the possibility to pause the test, for example, when you are interrupted by a phone call. Whereas a lack of face validity may hinder the use of DCAs, clear face validity would facilitate its use.

## Discussion

This study provides insight in the preferences of elderly and patients across the AD continuum for using DCAs. In this qualitative study we identified three main themes related to important questions that patients would ask themselves: (1) Do I want to know my brain health? (test motivation); (2) Do I want to use a *digital* test? (digital test suitability); (3) What makes a digital test a good test for me? (digital test characteristics). Within these themes several subthemes were identified. Of note, factors that were mentioned as facilitators for one person could be recognized as barriers by someone else. In the remainder we discuss important perspective differences and similarities, and provide recommendations based on this discussion.

Older adults and primary care patients have previously been demonstrated to be open towards cognitive testing,^33,34^ which was supported in our study by older individuals and memory clinic patients. Considerations regarding whether one wants to know one's brain health are inherently different depending on the place in the patient journey (i.e. healthy, worried and visiting memory clinic, or diagnosed). This was reflected in our observation that early disease recognition was only mentioned in the CU and SCD groups as a facilitator for test motivation. In all diagnostic groups, disease monitoring motivated test willingness, yet reasoning behind these motivators differed: For SCD participants, the wish for monitoring was driven by reassurance of unimpaired cognition (e.g. comparison with peers), whereas MCI participants wanted to monitor their cognition towards care planning.

An important barrier towards test willingness involved concerns of increased anxiety, as recognized previously by patients and health care professionals.^15,19,35^ A negative attitude towards cognitive testing caused by fear of dementia is also in line with previously described considerations for biomarker disclosure, concerning autonomy and justice such as the wish and/or right not to know.^36,37^ Although little is known about the actual impact of disclosing cognitive biomarkers in early disease stages, previous work has indicated that research participants feel they have the right to receive their test results, which can be perceived as personally valuable and empowering.^19,38^ Disclosing cognitive biomarkers may have similar impact as disclosing biological biomarkers. Literature on biological biomarker disclosure in preclinical stages has shown that individuals in early phases are welcoming to disclosure of disease status, which was perceived as emotionally safe, and could motivate research participation.^39–43^

The digital nature of DCAs poses the question whether the digital modality is appropriate for disclosing test results. Digital disclosure of test results was perceived both a facilitator and a barrier. Although we observed individual preferences on how to receive results, this may also depend on the context of use. For instance, the worried-well at home may prefer perceiving digital results themselves without a link to medical care, whereas patients who completed a DCA upon advice by their health care professional may prefer receiving results through the health care system. To meet individual preferences, it is advisable to personalize whether, when and how results are disclosed. For example, DCAs should provide the possibility to choose upfront whether results are disclosed directly or via a health care professional. In addition, as recognized previously,^17,44^ digital tests should provide a personalized diagnostic report to comprehensively summarize test results. Future work is needed for more insight in the best way to share cognitive performance results in early disease stages.

DCAs can improve efficiency and accuracy.^17,19,45^ Our participants also valued the additional benefit of DCAs to existing tests. Although it was not specifically discussed, we speculate that it depends on the purpose of the test whether DCAs can be a useful addition to traditional tests or a valid replacement of existing paper-and-pencil tests. Concerning DCAs that aim at early disease detection, for which an alternative test is currently not available, a useful addition regards an expansion of currently available tests. When related to DCAs that might replace a traditional neuropsychological assessment that patients perceive as burdensome,^46^ this factor may indicate that patients do not yet have trust in DCAs with regards to this purpose.

At-home testing and self-administration were regarded both a facilitator and barrier of DCAs. On the one hand, at-home testing could relieve both patient and caregiver burden, thereby saving time and costs. Self-administration might reduce test anxiety, and in the CU group specifically, anonymous testing before deciding upon seeking further medical help was raised as a facilitator. On the other hand, concerns were raised that the unstandardized home environment may be vulnerable to distractions from family members or pets, and requires self-discipline to complete the full test. In addition, a lack of personal contact was perceived as a barrier, as it is accompanied by increased negative emotions and fear of failure, aligning previous concerns.^17,19^

We recommend applying strategies to tackle barriers whilst exploiting facilitators. Based on the interplay between being motivated by insight in disease status, while impeded by the fear of disease progression, we recommend to pay attention to avoiding unnecessary worry when deciding on the use of DCAs. Modelled after guidelines for biomarker disclosure,^45,47^ the following steps towards using DCAs are recommended: (1) assessing whether cognitive testing is appropriate for a particular individual (i.e. What is the question in the context of care?); (2) delivering pretest education, ensuring understanding of implications and assessing motivation; (3) assessment; (4) returning results in clear language; and (5) providing for follow-up care.

To minimize distractions from the remote environment and mitigate technological barriers in self-administration, clear test instructions are essential, which could be accomplished by providing step-by-step tutorials^48^ and availability of (remote) technical support.^49^ Additionally, ensuring user-friendliness is essential, aligning previous observations.^17,18^ If gamification is used, it is crucial to stay aware that cognitive assessment remains a serious matter. Co-designing through active end user engagement can support in finding the optimal balance.^50^ Another aspect that touches upon user-friendliness considers test duration and frequency. Although lengthy testing was considered a barrier, as noted previously,^19^ some participants preferred one long test over multiple short tests across weeks or months, as continuous monitoring would induce feelings of distress. To meet individual preferences, we advise a personalized approach, aligning the previous recommendation of allowing for adaptations of functionalities to individual (cognitive) abilities.^48^ For example, our participants indicated personal preferences for adjustment of text size, provide context about for example fatigue or health problems, and possibilities to pause in case of long test duration. However, pausing options may not be feasible for all DCAs, as some tests require specific durations, such as tests for delayed recall. Additionally, the possibility to splitting a DCA into multiple sessions may affect its reliability, hindering comparison performance and computation of change. Hence, the optionality of pausing needs to be explored further, and should only be incorporated in a DCA if the specific test allows for this. Moreover, transparency on data usage is needed to reduce privacy concerns, a well-known barrier of digital testing,^9^ and transparency about the test provider and test validity would enhance trust in DCAs. For health care professionals trust in a test seems to mainly concern psychometric properties such as sensitivity/specificity and accuracy, or transparency about the possibilities and limitations of the test.^15,17^ Aligning our observations, for patients, trustworthiness is driven by perceiving the digital assessment as a valid measure of cognition and trust in the information source.^18,19^ Since DCAs from private companies might be perceived as less trustworthy, acknowledgement by renown universities or medical centres is crucial to overcome this barrier.

This study has several strengths and limitations. A strength of this study is the inclusion of older individuals in different cognitive stages, to explore the field of DCAs across different places in the patient journey. Another strength was that we succeeded in creating an environment where participants could both verbally and in writing openly share thoughts and feelings. Nevertheless, our study has some limitations. Firstly, group sizes were moderate and most participants were highly educated, which could possibly limit the generalizability. Still, we succeeded to obtain insights from diverse perspectives by including participants from a broad age range (58–85), and both from clinical research cohorts and the general population. Secondly, particularly in the MCI group, hypothetical reasoning could be difficult, and some uncertainty remains about whether all important topics were properly discussed. To anticipate, we explained the concept of DCAs using concrete examples, and all participants were familiar with touchscreen-based devices, which improved the understanding of DCAs.

Future research is needed to deepen our work. Some of the raised barriers and facilitators may specifically apply to the context of use. For each context of use a different type of DCA may be the most appropriate. However, in case that different DCAs are used in practice, harmonization procedures for test comparison are necessary. To illustrate, we speculate that for the worried-well and screening and diagnostic purposes, a single test could be used to reassure normal cognition or make a diagnosis, while for monitoring purposes continuous passive testing might be better suited. Future research should identify facilitators and barriers for specific contexts of uses, and determine what DCA type fits each context of use best. In this regard, research is needed to explore who (e.g. patient or health care professional) should initiate DCA, which again may depend on the context of use. Moreover, other applications, such as assistance in daily life to maintain functioning, or information provision about intervention strategies, were briefly touched upon during focus groups, but were disregarded as this topic was out of scope of the focus groups. Further research should address the needs and wishes regarding tools serving such applications.

In conclusion, older individuals and patients recognized the potential of DCAs. Multiple facilitators and barriers were identified for use. Importantly, what could be a facilitator for one might hinder someone else, underscoring the need for personalization in the use of DCAs. Such a personalized approach could foster their large-scale implementation.

## Supplemental Material

sj-docx-1-dhj-10.1177_20552076251318903 - Supplemental material for Focus groups on digital cognitive assessment in the context of Alzheimer's disease Supplemental material, sj-docx-1-dhj-10.1177_20552076251318903 for Focus groups on digital cognitive assessment in the context of Alzheimer's disease by Sophie M van der Landen, Rosanne L van den Berg, Matthijs J Keijzer, Mariska N van Liere, Casper de Boer, Leonie N C Visser, Wiesje M van der Flier, Hanneke F M Rhodius-Meester and Sietske A M Sikkes in DIGITAL HEALTH
