# Supplementary material for: Focus groups on digital cognitive assessment in the context of Alzheimer's disease
Source: Digit Health. 2025 Feb 12;11:20552076251318903. doi: 10.1177/20552076251318903 (PMC11815793; doi:10.1177/20552076251318903)
Supplement: sj-docx-1-dhj-10.1177_20552076251318903 - Supplemental material for Focus groups on digital cognitive assessment in the context of Alzheimer's disease [file sj-docx-1-dhj-10.1177_20552076251318903.docx]

**Supplementary Table 1.** Themes and identified facilitators and barriers by diagnostic group.

|  | **Theme** | **CU** | **SCD** | **MCI and care partners** |
| --- | --- | --- | --- | --- |
| **1** | **Test motivation** |  |  |  |
| 1.1 | Early disease recognition | Facilitator | Facilitator | NA |
| 1.2 | Monitoring performance over time | Facilitator | Facilitator | Facilitator |
| 1.2.1 | Compare with peers | NA | Facilitator | NA |
| 1.2 | Fear of dementia diagnosis | Barrier | Barrier | Barrier |
| 1.3 | Own sense of decline | NA | Barrier | Barrier |
| **2** | **Digital test suitability** |  |  |  |
| 2.1 | Saves time and health costs | NA | Facilitator | NA |
| 2.2 | Useful addition to existing tests | Facilitator | Facilitator | NA |
| 2.3 | Digital test result | Facilitator  Barrier | Facilitator  Barrier | Facilitator  Barrier |
| 2.4 | Linked to care | Facilitator  Barrier | Facilitator | NA |
| 2.4.1 | Anonymous | Facilitator | NA | NA |
| 2.4.2 | Lack of personal contact | Barrier | Barrier | NA |
| 2.4.2.1 | Negative thoughts (fear of failure) | Barrier | Barrier | NA |
| 2.5 | Self-administration | Facilitator  Barrier | Facilitator  Barrier | Barrier |
| 2.5.1 | Self-discipline | Barrier | Barrier | Barrier |
| 2.6 | At home | Facilitator  Barrier | Facilitator  Barrier | Facilitator  Barrier |
| 2.6.1 | At your convenience | Facilitator | Facilitator | Facilitator |
| 2.6.2 | Less burdensome | Facilitator | Facilitator | NA |
| 2.6.3 | Distractions | Barrier | Barrier | Barrier |
| **3** | **Digital test characteristics** |  |  |  |
| 3.1 | User-friendliness | Facilitator | Facilitator | Facilitator |
| 3.1.1 | Attractive interface | Facilitator | NA | Facilitator |
| 3.1.2 | Gamification | Facilitator  Barrier | NA | Facilitator  Barrier |
| 3.1.2.1 | Childish | Barrier | NA | Barrier |
| 3.1.2 | Clear test instructions | Facilitator | Facilitator | NA |
| 3.3 | Thrustworthy test | Facilitator | Facilitator | NA |
| 3.3.1 | Face validity | Facilitator | Facilitator | NA |
| 3.3.2 | Physical obstacles | Barrier | Barrier | NA |
|  |  |  |  |  |
| 3.5 | Test duration | Facilitator  Barrier | Facilitator  Barrier | Facilitator  Barrier |
| 3.3.3 | Providing context | Facilitator | Facilitator | NA |
| 3.7 | Aversion to digital world | Barrier | Barrier | Barrier |
|  |  |  |  |  |
| 3.7.1 | Privacy | Barrier | Barrier | NA |
| 3.7.2 | Digital incompetence | Barrier | Barrier | Barrier |

*Note.* CU = cognitively unimpaired, SCD = subjective cognitive decline, MCI = mild cognitive impairment. NA indicates not applicable (i.e., factor is not raised as facilitator or barrier).


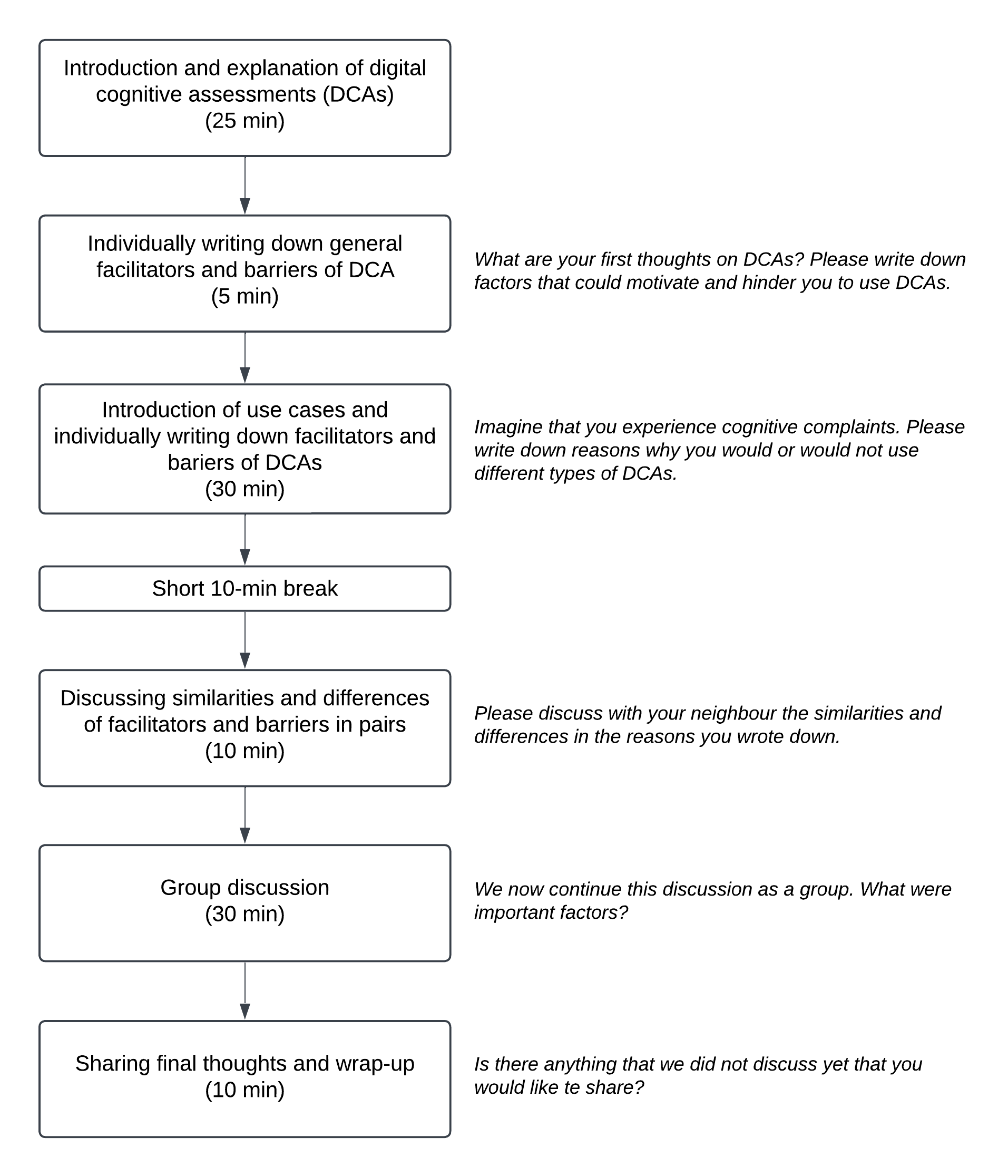


**Supplementary Figure 1.** Focus group procedure and interview guide.
